# Applicability of an Active Back-Support Exoskeleton to Carrying Activities

**DOI:** 10.3389/frobt.2020.579963

**Published:** 2020-12-09

**Authors:** Tommaso Poliero, Maria Lazzaroni, Stefano Toxiri, Christian Di Natali, Darwin G. Caldwell, Jesús Ortiz

**Affiliations:** ^1^Department of Advanced Robotics, Istituto Italiano di Tecnologia, Genoa, Italy; ^2^Department of Informatics Bioengineering Robotics and Systems Engineering, University of Genoa, Genoa, Italy; ^3^Department of Electronics, Information and Bioengineering, Politecnico di Milano, Milan, Italy

**Keywords:** exoskeleton, occupational exoskeleton, versatility, lifting, carrying, task recognition, human activity recognition

## Abstract

Occupational back-support exoskeletons are becoming a more and more common solution to mitigate work-related lower-back pain associated with lifting activities. In addition to lifting, there are many other tasks performed by workers, such as carrying, pushing, and pulling, that might benefit from the use of an exoskeleton. In this work, the impact that carrying has on lower-back loading compared to lifting and the need to select different assistive strategies based on the performed task are presented. This latter need is studied by using a control strategy that commands for constant torques. The results of the experimental campaign conducted on 9 subjects suggest that such a control strategy is beneficial for the back muscles (up to 12% reduction in overall lumbar activity), but constrains the legs (around 10% reduction in hip and knee ranges of motion). Task recognition and the design of specific controllers can be exploited by active and, partially, passive exoskeletons to enhance their versatility, i.e., the ability to adapt to different requirements.

## 1. Introduction

In the 1970s, the scientific community began addressing the relationship between musculoskeletal disorders (MSDs) and work ergonomics. Since then, many studies have been published regarding this topic (Bernard and Putz-Anderson, 1997; Cohen, 1997; Fujishiro et al., 2005; Hamberg-van Reenen et al., 2008). Yet, in the most recent EU-OSHA report de Kok et al. (2019), MSDs are still cited as the most common work-related health problem in the EU. Indeed, 60% of workers still experience such disorders, in the majority of the cases due to back pain. MSDs affect not only the workers, but also the enterprises that, in turn, have to cope with absenteeism and productivity losses. To have an idea of the economic impact, in 2012, the total annual cost related to MSDs to the European Community represented 2% of the GDP (Bevan, 2012).

Workers performing manual material handling (MMH) activities (e.g., package loading and unloading in a warehouse or luggage handling in airports) are among the most exposed to risks and injuries. To try to reduce MSDs associated with MMH, NIOSH has developed a method for the ergonomic assessment of a task, defining whether or not it is classified as risky (Waters et al., 1993). Potentially harmful tasks should be mitigated via adoption of different solutions such as the introduction of limits for handled masses, frequencies, and task duration. Additionally, companies have tried to mitigate MSDs by re-designing the workplace according to the newer ergonomic guidelines or by resorting to plant automation and to the introduction of industrial manipulators. However, the cost associated with these solutions and the lack of adoption of external tools by the users prevents the problem of MSDs from being completely solved.

### 1.1. Back-Support Exoskeletons and Lifting

The ability of back-support exoskeletons to reduce the physical loading on the lumbar spine while performing lifting tasks suggests that they may present a possible novel solution to back pain-related MSDs. Indeed, a 2016 review on occupational exoskeletons reported that usage of back-support exoskeletons yielded a 10–40% reduction in back muscle activity during repetitive lifting and static holding tasks (de Looze et al., 2016). The primary consequence of muscle activity reduction is the de-compression of the lumbar spine. Such results are confirmed by a more recent review (Theurel and Desbrosses, 2019) that stresses the clear potential of exoskeletons in limiting muscular demand. However, this report also warns that there is insufficient current knowledge to justify an unreserved adoption of this technology. Fox et al. (2019) elaborate on these devices to improve manufacturing processes. Moreover, focusing on three aspects, namely (a) actuators, (b) structures and physical attachments, and (c) control strategies employed, Toxiri et al. (2019) report on the technical development of back-support exoskeletons meant for occupational use. According to the actuator choice, an exoskeleton can be defined as active or passive. A passive exoskeleton exploits its wearer's movements to store and then release energy. Energy storage is achieved by means of passive elements such as gas/coil springs, flexible beams or elastic bands (Abdoli-e et al., 2006; Lamers et al., 2017; Näf et al., 2018). In contrast to passive exoskeletons, active devices have the ability to deliver additional energy to the user exploiting electrical motors or pneumatic actuators. Such active elements, rather than relying onto the users' movement, are powered by batteries or external supplies. Properly controlling the active actuators allows designers to tune the assistance being provided based on different control strategies. As an example, in Toxiri et al. (2018) and Tan et al. (2019) sEMG signals are used to modulate the assistive torque, while in Lazzaroni et al. (2020), Chen et al. (2018), Ko et al. (2018), Zhang and Huang (2018), and Yu et al. (2015) the control relies on kinematics.

### 1.2. Manual Material Handling: Is There Only Lifting?

As reported in Grazi et al. (2019), a consensus on the methods and metrics for the evaluation of back-support exoskeletons is still lacking. Indeed, the analyzed signals, the testing conditions, and the performance metric vary across the many available studies. However, all these studies have in common that the exoskeleton evaluation only considers static bending and symmetric lifting tasks. Yet, risk of overload for workers arises not only from lifting: workers may find themselves performing many different activities in the same workplace. As an example, in logistics, it is possible to imagine a quite simple task where a worker *walks* to the shelf, *picks* the required object, *carries* it back to the cargo area, and, eventually, *lowers* it in the appropriate container. A similar scenario can be pictured in other contexts where MMH is involved. In such conditions, the International Standard ISO 11228 establishes ergonomic guidelines not only for lifting but also carrying, pushing, and pulling. Therefore, the analysis of the exoskeleton usage effects should not be limited to lifting tasks, but importantly should also tackle other activities such as carrying, pushing, pulling, and walking. This extension can capture the complexity of *out-of-the-lab* environments more reliably. As an example, an interesting study presented in Baltrusch et al. (2019) focuses on the versatility of a passive exoskeleton, studying its performance not only related to lifting but also walking. As might be expected, it emerges that passive exoskeletons provide benefits during lifting and do restrict the movement during walking. From this point of view, active exoskeletons, even if more complex and heavier, are expected to perform better, because of the possibility of tuning and customizing the assistance according to the task.

### 1.3. Contribution of This Study

Recent works on exoskeletons have discussed about the opportunity of exploiting human activity recognition to discriminate between different tasks such as lifting, walking, carrying, or sitting (Chen et al., 2018, 2019; Poliero et al., 2019a; Jamšek et al., 2020). For passive exoskeletons, this implies that, by using clutches for the engagement and disengagement of passive elements, as in Endo et al. (2006), Walsh et al. (2007), Ortiz et al. (2018), Jamšek et al. (2020), and Di Natali et al. (2020a), it is possible to assist only when needed, i.e., deactivate the passive elements when they create a restriction such as in the walking case. Active exoskeletons, on the other hand, thanks to their actuators versatility, could implement specific controllers for any of the previous tasks.

In the study presented hereafter, the investigation focuses on carrying activities, given their relevance to MMH and to the ISO 11228-1 standard. In particular, the authors want to elaborate more on (i) *the impact that a non-lifting activity might have on lower-back loading* and on (ii) *the need to select different controllers based on the performed task*.

First, a comparison is made between the spinal loading during lifting and carrying activities to investigate the impact of the task on this latter parameter. In particular, spinal loading, which is closely associated with risk of injuries, is caused by the activation of deep back muscles—related to back extension—generating compression on lumbar discs. When a worker is carrying a load, back extensors activate to keep the trunk stable and straight, thus, this situation also presents risks to the user.

Second, to better understand the need of different controllers according to the task, it might be useful to report a consideration. To date, the vast majority of available occupational back-support exoskeletons are designed and programmed to provide assistive torques that contribute *simultaneously* to the extension of the back and both hips, regardless of their actuation principles and control strategies. This assistance principle is derived from and replicates the typical movements observed during symmetric lifting activities. Indeed, in this situation, every time there is back flexion or back extension, there is also a corresponding flexion or extension of the hips, respectively. Therefore, the presented assistance principle seems appropriate. In this study, the soundness of applying this strategy in carrying activities is investigated. Indeed, the inclusion of gait shows a different situation with respect to symmetric lifting. In particular, during carrying, contributing to back extension is appropriate, but simultaneously pushing both hips toward extension might interfere with their natural movement. More specifically, the support could be beneficial during hip extension (associated with the leg in stance phase), but may result in restriction of the hip flexion (forward swing, characteristic of the leg not in contact with the ground). Hence, to understand the need of different controllers according to the task, the effects that assisting with carrying—adopting an assistance principle derived from observation of symmetric lifting activities—has on the users are studied. The effects will be assessed in terms of muscle activity, gait kinematics, and subjective perceptions.

In the following, details on how the experimental testing was devised are reported in Section 2. Section 3 presents the results that are then discussed in Section 4. Finally, Section 5 summarizes and concludes this work.

## 2. Materials and Methods

We devised an experiment, approved by the Ethics Committee of Liguria^1^, that is detailed following the description of *XoTrunk*, the active back-support exoskeleton used in this study. Finally, information on data processing and outcome measures are reported.

### 2.1. XoTrunk: An Active Back-Support Exoskeleton

**Figure 1:**
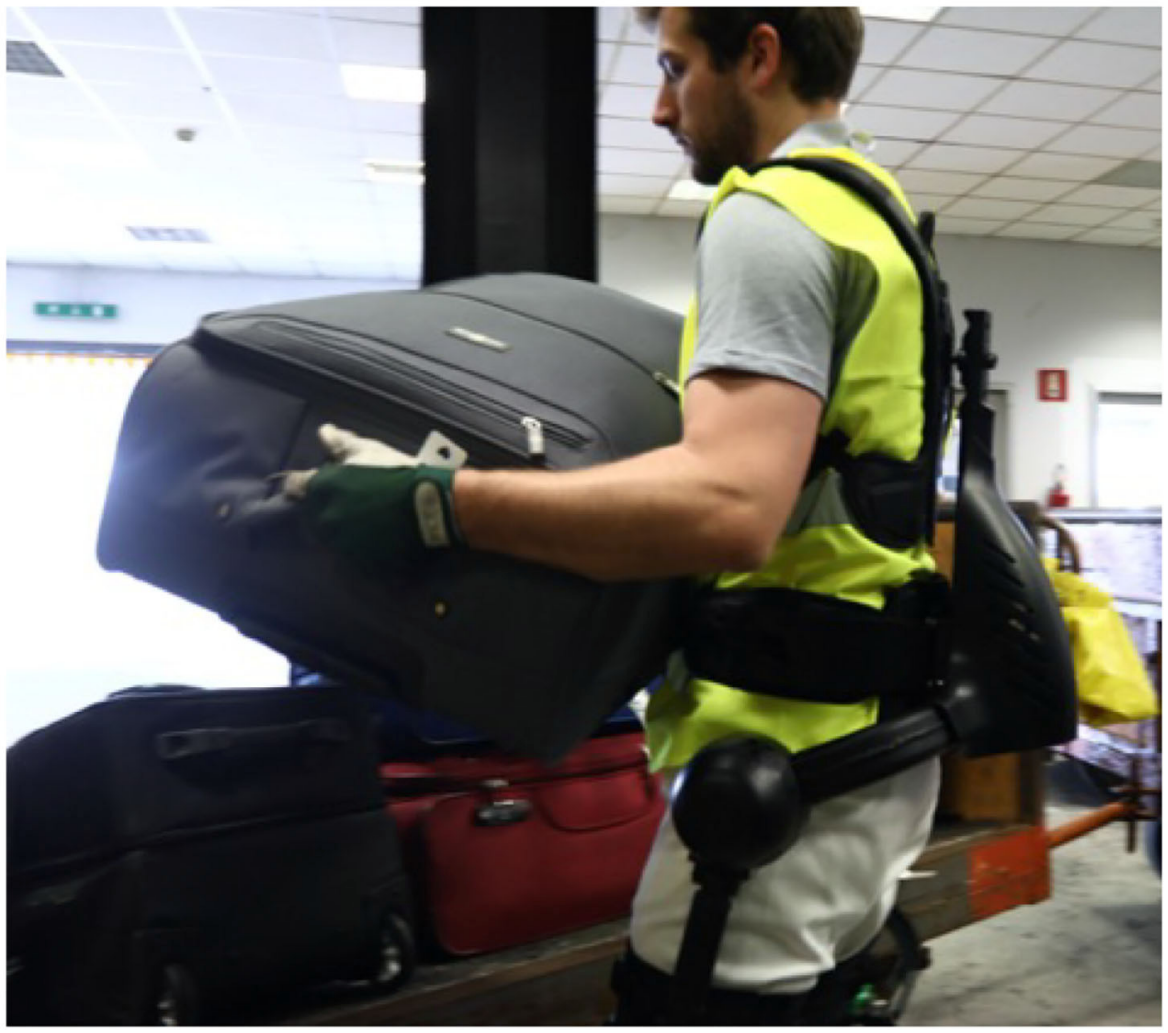
Example of luggage handling in an airport performed with the usage of XoTrunk. Written informed consent was obtained from the individual pictured in the figure.

XoTrunk (see Figure 1) is a 6kg improved version of the Robo-Mate prototype, presented in Toxiri et al. (2018). Its aluminum frame houses the control and electronics box, the actuation units, and the anchoring points. These points are situated close to the thighs and the shoulders, allowing the device to transmit the torques—produced by its two brushless DC motors—to the wearer. These torques are used to help the user perform lifting, by partially contributing to hip and back extension. Additional anchoring on the waist provides more stability and comfort. More details on the actuators and low level control can be found in Di Natali et al. (2020b), whereas kinematics and physical attachments are reported in Sposito et al. (2020).

The versatility provided by its two electrical motors allows to test and study different control strategies. In particular, in Toxiri et al. (2018), three control strategies are presented to modulate the torque proportionally to (a) the torso inclination angle, (b) the forearm muscle activity, and (c) a combination of torso inclination and forearm muscle activity. Regardless of the selected control strategy, the motors always provided assistive torques that contribute simultaneously to the extension of the back and both hips. The backwards push on the back is the combination of the assistance provided by the left and right sides. As introduced in Section 1.3, this assistance principle is inspired by observation of symmetric lifting movements. This study concerns whether or not this assistance principle can be beneficial also for assisting carrying. The control strategy selected here was based on a constant extension torque provision. Indeed, for the sake of simplicity, during carrying the torso inclination can be neglected, whereas the forearm muscle activity can be assumed to be constant during load handling. Such simplifications were introduced to facilitate the analysis of the effects that assistance during carrying has on the users. In the following, this control mode is referred to as the Exoskeleton On (*Exo-on*) condition. Each motor generates a constant torque of 10 Nm, resulting in an overall assistance of 20 Nm.

### 2.2. Experimental Set-Up and Protocol

Nine healthy male subjects (*N* = 9, 1.78 ± 0.04 m, 76.55 ± 8.22 kg, 31 ± 3.46 years old) were asked to wear sporting clothes and informed they would have to perform the following tasks:

- *Lifting*: The sequence of: standing upright, reaching for a box lying 0.30 m from the ground, grasping and lifting it, reaching upright posture again, then putting the box back down on the ground and returning to the upright posture. Each sequence was repeated three times at a self-selected speed and with a freestyle lifting technique, meaning no specific instructions on lifting motion were given (Burgess-Limerick, 2003).
- *Carrying*: Straight level walking for 7.5m, while holding a box close to the trunk at self-selected speed.

Each test subject performed lifting with the box (1.2 kg) housing three different payloads, namely 0, 7, and 15 kg. In the following, the different weights are referred to as *light* (*L*), *medium* (*M*), and *heavy* (*H*). All the conditions were repeated three times for a total of 9 tests per subject. Carrying tasks were performed not only varying the loads (light, medium and heavy, as for lifting), but also the supplied assistance. In particular, two conditions were tested:

a) No Exoskeleton (*No-exo*): carrying without the exoskeleton;
b) Exoskeleton On (*Exo-on*): carrying while wearing the exoskeleton in the *on-mode*. The exoskeleton provides an angle independent constant torque of 20 Nm to provide support for the extension of the back and of both hips (see Section 2.1).

Each load and assistance condition was repeated three times for a total of 18 tests per subject. The task execution order, the handled weights, and the supplied assistance were randomized between subjects.

At the end of the experimental protocol, the subjects were asked to fill in a simplified version of an RPE (Rate of Perceived Exertion) questionnaire to rate the differences between carrying in the *No-exo* and in the *Exo-On* condition (Huysamen et al., 2018).

Table 1 summarizes the protocol and its independent variables.

**Table 1:** Overview of the testing protocol and the selected metrics.

Tasks	Lifting; Carrying
Loads	Light 1.2 kg (*L*), Medium 8.2 kg (*M*), Heavy 16.2 kg (*H*)
Conditions	Without exoskeleton (*No-exo*); with exoskeleton (*Exo-on*)
Repetitions	3x
Metrics	*M*, *P*, *RoM*_*h*_, *RoM*_*k*_, $\bar{\delta }$
Statistical analysis	for each ρ^*x*^, α, iqr, and γ were analyzed (*x* being any of the above metrics)

### 2.3. Measurements and Data Processing

**Figure 2:**
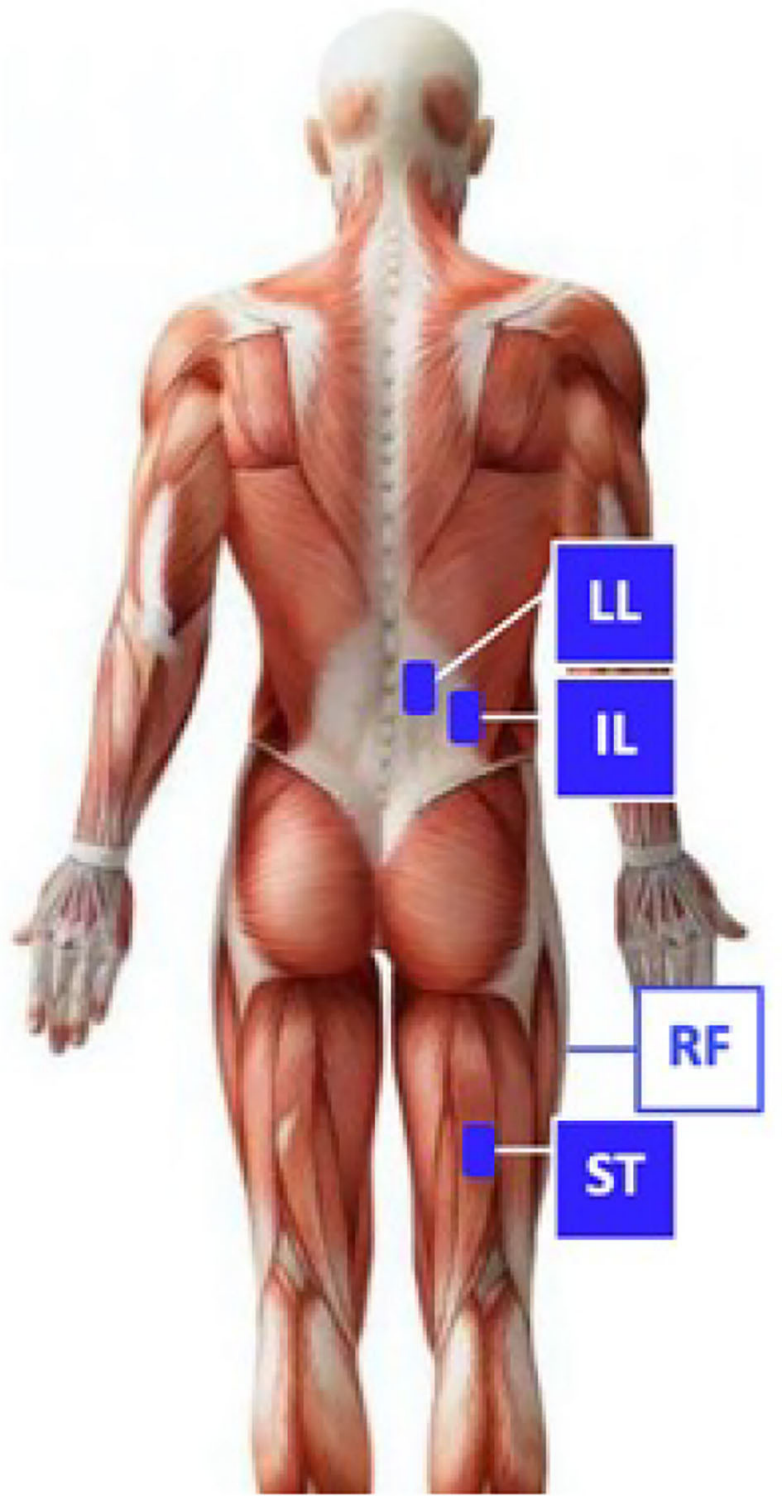
Schematic representation of electrodes placement. For greater clarity, only the right side electrodes are displayed for the back. It is possible to identify the Erector Spinae Longissimus Lumborum (LL), the Erector Spinae Iliocostalis (IL), the rectus femoris, and the semitendinosus.

To collect muscular activity data, the subjects were asked to wear surface EMG (sEMG) electrodes (BTS FREEEMG, BTS Bioengineering, Italy). These latter were placed, according to SENIAM guidelines, to measure the bilateral activation of the muscles responsible for trunk extension, namely the Erector Spinae Longissimus Lumborum (LL) and the Erector Spinae Iliocostalis (IL). Additionally, due to the symmetry of the task, only the subjects' right leg was instrumented to measure the activation of two muscles responsible for hip flexion and extension, i.e., the rectus femoris (RF) and the semitendinosus (ST). Back and leg muscles were chosen based not only on their relevance when performing lifting activities but also on the number of studies that analyze them in order to allow comparisons of findings across different protocols (Grazi et al., 2019). Figure 2 illustrates the locations of the chosen muscles. Prior to attaching the electrodes onto the skin, the site was cleaned with alcohol, as suggested in Stegeman and Hermens (2007). Muscular activity information was acquired at a sampling frequency of 1 kHz. Extraction of metrics from the sEMG signals requires data post-processing. The common approach reported in Pons (2008) consists of filtering the amplified raw sEMG signals (BTS FREEEMG output), rectifying the output, and, eventually, computing the linear envelope (low-pass frequency filter at 2.5 Hz, Potvin et al., 1996). EMG data were normalized to maximum voluntary contractions (MVC) (McGill, 1991). Overall, lumbar extensor activity (averaged IL and LL muscle activity, right and left side) was computed prior to performing deep-back muscles analysis as in Koopman et al. (2019).

To collect motion data, the subjects were also equipped with a 3D motion tracking system (MTw Awinda, Xsens, The Netherlands). 7 Xsens IMUs were attached to the feet, shanks, thighs, and pelvis in order to reconstruct lower limb kinematics and gait phase events. The Xsens software can reconstruct motion data at a 60Hz sampling frequency. Using IMU trackers and biomechanical models, the software also provides gait phase information that can be used to perform data segmentation (Di Natali et al., 2020a). Two consecutive *heel strike* events generated by the same foot are used to identify the start and finish of the stride.

Before data recording, Xsens calibration and MVC acquisition routines were performed for each subject (Vera-Garcia et al., 2010; Halaki and Ginn, 2012).

### 2.4. Outcome Metrics and Analysis

In the following, the metrics used for the assessment of the effects of assisting with carrying are reported along with the metrics used for comparing carrying and lifting tasks. This section also introduces how the statistical analysis was performed. Table 1 summarizes what presented hereafter.

#### 2.4.1. The Effects of Assistance During Carrying

As previously introduced in Section 1.3, it is hypothesized that the exoskeleton will positively influence the back and hips extension, whereas the hip flexion would be hindered. To explore the effects of assisting with carrying, this task was analyzed in the *No-exo* condition (control group) and in the *Exo-on* state (test group). To be consistent with studies focusing on lifting, the effect of the exoskeleton on the back is analyzed in terms of muscle activation. For the lower limbs, on the other hand, gait inclusion suggests also adding gait kinematics analysis to the muscle activation. In the following, first the muscle analysis metrics are presented and, then, the gait kinematics are considered.

Muscle fatigue may be experienced as symptoms or signs of reduced motor control such as localized discomfort or decreased strength. Generally, physical exertions can cause fatigue that lasts for just a few hours. If fatigue persists, it may cause tissue damage and yield MSDs (ACGIH, 2008). In Jonsson (1982), the 50th percentile/median of the muscle activity distribution (*M*) is selected to reflect how the muscle has been working during the whole recording period. Based on this reasoning, in this work, *M* was chosen to monitor the risk associated with repetitive/cumulative fatigue both for the back and the lower limb muscles. Additionally, ergonomic guidelines for industry define the maximum allowed spinal compression. If this threshold is exceeded, traumatic damages in the inter-vertebral discs may result (Moore and Garg, 1995). Biomechanical models can be used to show how this compressive force is directly linked to muscle activity (Chaffin, 1969; Toxiri et al., 2015). In Jonsson (1982), the 90th percentile of muscle activity distribution (*P*) is indicated as being more informative than the maximum muscle activity. For such reasons, in this work, *P* was chosen to monitor the risk associated with traumatic damages in the inter-vertebral discs. *P* was analyzed also for the lower limb muscles, even though there is no clear traumatic damage associated with those sites.

The gait kinematics is focused on the hip and knee ranges of motion (*RoM*_*h*_ and *RoM*_*k*_, respectively) that are defined as the difference between the 90th and the 10th percentile of the lower limbs trajectory distribution during carrying. Since users were instructed to walk at a self-selected speed, an analysis on the average stride time ($\bar{\delta }$) per condition is conducted. $\bar{\delta }$ is defined as in Equation (1)

(1) $$\bar{\delta }=\frac{1}{S-1}\sum_{k=1}^{S-1}H_{k+1}-H_{k},\text{ for k }=1,2,...,\text{S-}1$$

where *S* represents the number of strides in a test and *H* is a vector collecting all the right heel strike time events.

#### 2.4.2. Comparing Carrying and Lifting Tasks

To report on the impact that carrying has on spinal loading compared to lifting, a simple comparison of lifting in the No-exo condition (control group) and carrying in the No-exo one (test group) is presented. This analysis was focused on the overall lumbar extensor activity and on the same metrics presented in Section 2.4.1.

#### 2.4.3. Statistical Analysis

Kinematic data were analyzed applying a standard one-way analysis of variance (ANOVA) with significance level set at *p* < 0.05. Such analysis was performed for both hip and knee angles. Initially, the same approach was meant to be adopted also for the stride duration examination and the muscle activity one. However, due to large variability in inter-subject walking speed and muscle activation signals (even after normalization with respect to the MVC), the choice was made to center the analysis around intra-subject variability. Indeed, big data variability implies that standard statistical analysis would not be very informative. For this reason, ratios between the test and control conditions (specified in Sections 2.4.1, 2.4.2) were adopted as an alternative form of intra-subject normalization, prior to comparison with the results obtained by other subjects. In the following, we define $\rho_{i}^{x}$ as the ratio computed considering metric *x* (either *M*, *P*, *RoM*_*h*_, *RoM*_*k*_, or $\bar{\delta }$) in the control and test condition for a subject *i*.

(2) $$\rho_{i}^{x}=\frac{X_{i}^{control}}{X_{i}^{test}}$$

The vector collecting $\rho_{i}^{x}$ for all the nine healthy subjects is referred to as ρ^*x*^. To deepen the analysis of ρ^*x*^, and to better highlight trends in the data, the following values are taken into account for each ρ^*x*^ distribution:

- the median value (α);
- the inter-quartile range (iqr), defined as the difference between the 75th and the 25th percentile of the ρ^*x*^ distribution;
- the number of subjects for which $\rho_{i}^{x}<1$ (γ).

#### 2.4.4. Subjective Evaluation

The subjective evaluation forms, filled in by each subject at the end of the experimental protocol, allow a comparison to be made on whether or not the perceived effect is consistent with the objective data. Based on their relevance in this study, only the answers referring to back, waist, and legs are analyzed.

## 3. Results

In the following, results referring to spinal loading during carrying are presented, followed by those associated with the effects of the assistance during carrying. In particular, these latter results are split into muscle analysis and gait kinematics.

### 3.1. Spinal Loading During Carrying

**Figure 3:**
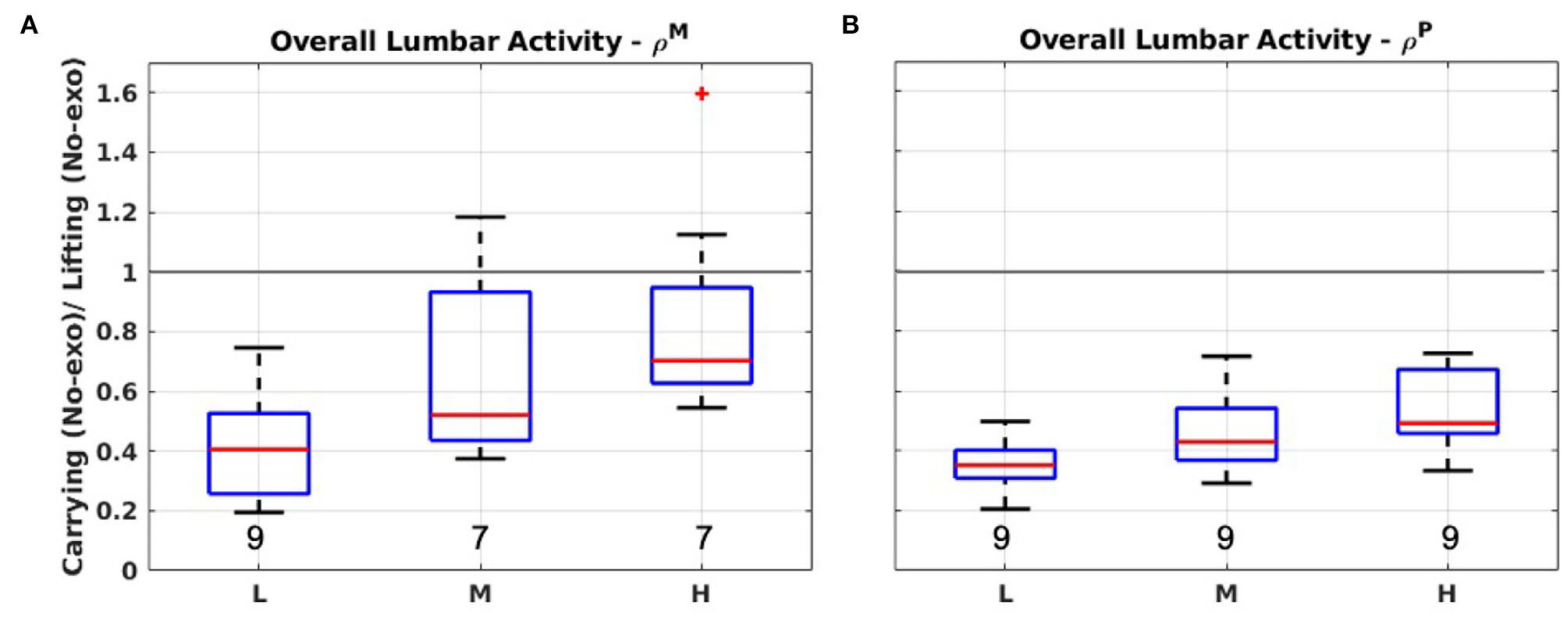
Boxplot representations for ρ^*M*^ and ρ^*P*^ considering the overall lumbar muscle activity performing lifting and carrying in *No-exo* condition. **(A)** The 50th percentile of muscle activation (ρ^*M*^). **(B)** The 90th percentile (ρ^*P*^). L, M, and H refer to the light, medium, or heavy loading condition. A gray line identifies where ρ = 1 (i.e., where carrying and lifting are equivalent according to the selected metric). Numeric values at the bottom of each box report the value of γ.

Figures 3A,B present the boxplot of the distribution of ρ^*M*^ and ρ^*P*^ when comparing the overall lumbar muscle activity during carrying and lifting activities.

Lower-back muscle activation is in the same order of magnitude, but generally lower during carrying compared to lifting, according to the reported measurements. This is true in all cases for ρ^*P*^, whereas ρ^*M*^ shows a few subjects for which $\rho_{i}^{M}>1$, meaning that the lumbar muscle median activation (50th percentile) was higher in carrying than lifting. In the heavy load test, one of the subjects is considered an outlier (represented by a red cross). For light loads, considering both ρ^*M*^ and ρ^*P*^, the median (α) is around 0.40, while this number increases to around 0.60 for heavier loads, showing an overall trend. It is worth highlighting that γ is always quite close to *N* = 9, indicating a shared trend among all the subjects.

### 3.2. Effects of Assistance During Carrying

The results are reported focusing first on the muscle activation and, then, on the gait kinematics.

#### 3.2.1. Muscle Activation

**Figure 4:**
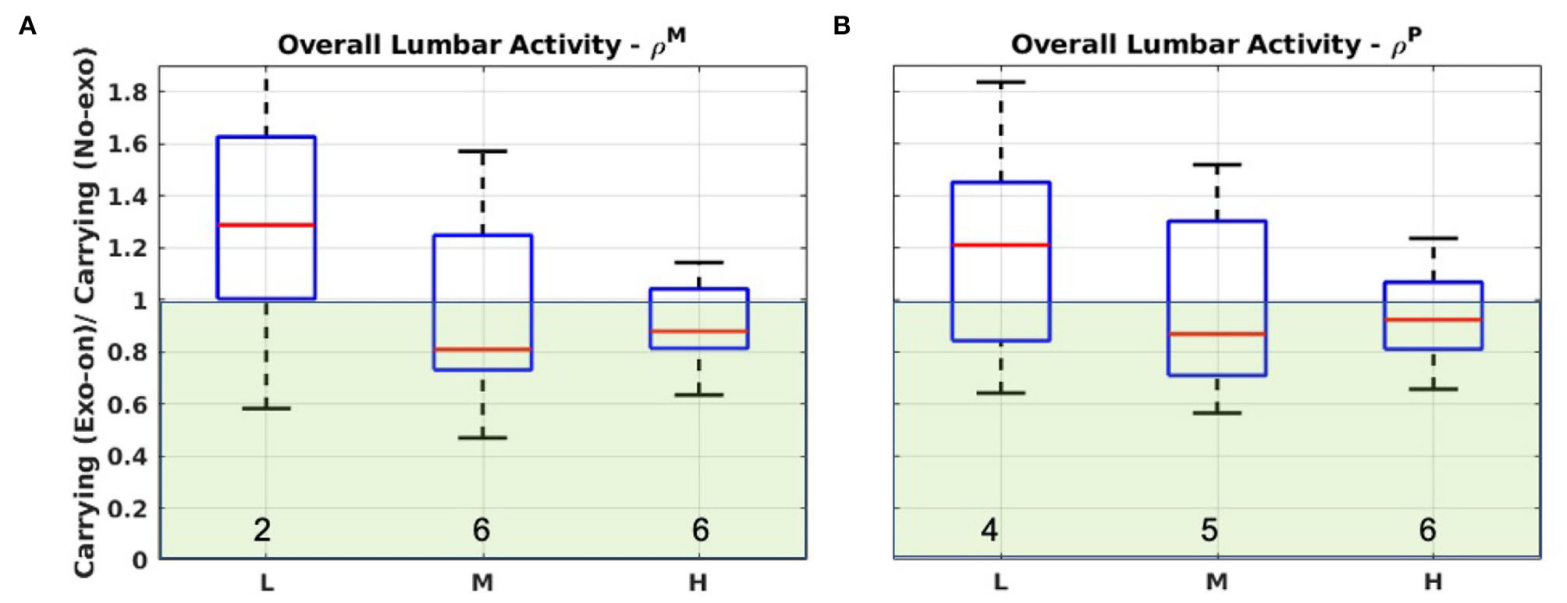
Boxplot representations for ρ^*M*^ and ρ^*P*^ considering the overall lumbar muscle activity performing carrying in the *No-exo* condition and in the *Exo-on* one. **(A)** The 50th percentile of muscle activation (ρ^*M*^). **(B)** The 90th percentile (ρ^*P*^). L, M, and H refer to the light, medium, or heavy loading condition. Green-shaded areas identify the regions where $\rho_{i}^{x}<1$ (i.e., the exoskeleton reduces the muscle activity). Note that in **(A)** the top whisker for the light loading condition extends up to 2.3. Numeric values at the bottom of each box report the value of γ.

Figure 4 reports the boxplot associated with ρ^*M*^ and ρ^*P*^ for the overall activation of the lumbar muscles when comparing carrying activities with and without the exoskeleton. Overall, the population distributions are around the unit value and the *iqr* range is quite large (up to 0.62). However, the *iqr* has a trend to reduce as the payload increases, both for mean and peak. Indeed, the variability in the heavy load condition is about one-third of that recorded for the lighter loads.

Light and heavy load tests display opposite behaviors, with the first (light load) belonging almost entirely to the ρ^*x*^ > 1 region (i.e., the exoskeleton produced an increase of the metric) and the second (heavy load) to the ρ^*x*^ < 1 region (i.e., the exoskeleton produced a reduction of the metric). This is more evident for ρ^*M*^ rather than ρ^*P*^. An additional interesting observation is that for both metrics the lowest value is for the intermediate weight. For both ρ^*M*^ and ρ^*P*^, γ indicates that the majority of the subjects experienced a reduction of muscle activation in the *Exo-on* condition, with respect to the control case. Moreover, as the payload increases, the value of γ increases as well.

**Figure 5:**
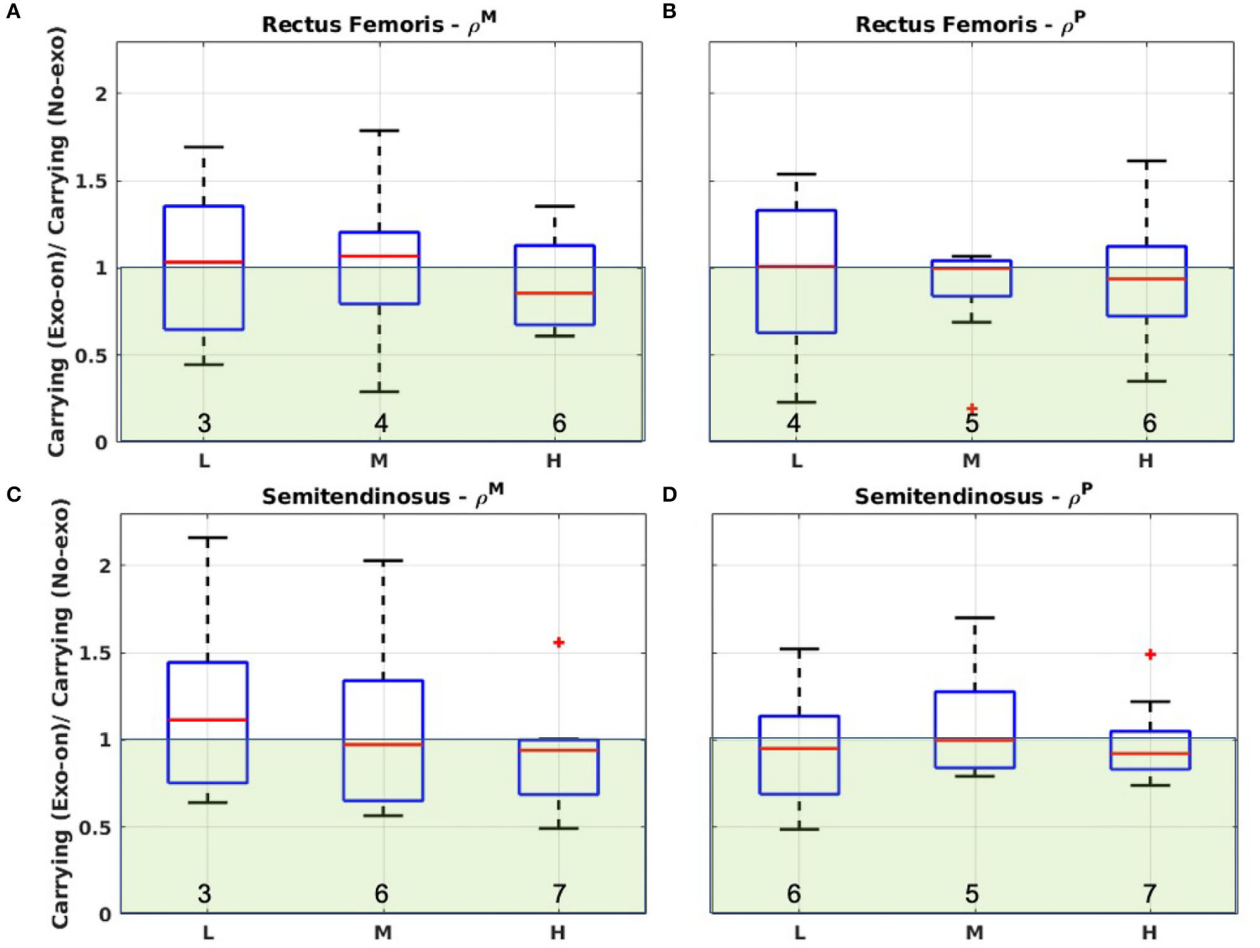
**(A)** Boxplot of ρ^*M*^ considering the rectus femoris activity performing carrying in *Exo-on* and *No-exo* condition. **(B)** Boxplot of ρ^*P*^ considering the rectus femoris activity performing carrying in *Exo-on* and *No-exo* condition. **(C)** Boxplot of ρ^*M*^ considering the semitendinosus activity performing carrying in *Exo-on* and *No-exo* condition. **(D)** Boxplot of ρ^*P*^ considering the semitendinosus activity performing carrying in *Exo-on* and *No-exo* condition. L, M, and H refer to the light, medium, or heavy loading condition. Green-shaded areas identify the regions where $\rho_{i}^{x}<1$ (i.e., the exoskeleton reduces the muscle activity). Numeric values at the bottom of each box report the value of γ.

Figure 5 refers to the lower limb muscles activation analysis. Similarly to above, the distributions are centered around the unit value. The *iqr* still displays large variability (up to 0.71) and there is no longer a clearly narrowing trend as the payload increases. Indeed, in the case of the RF, the *iqr* is smaller for the intermediate loads than it is for heavier loading condition. Red crosses identify outliers in the ST ρ^*M*^, and in the RF and ST ρ^*P*^. Also in this case, it is possible to identify an increasing trend for γ as the payload increases.

#### 3.2.2. Gait Kinematics

**Figure 6:**
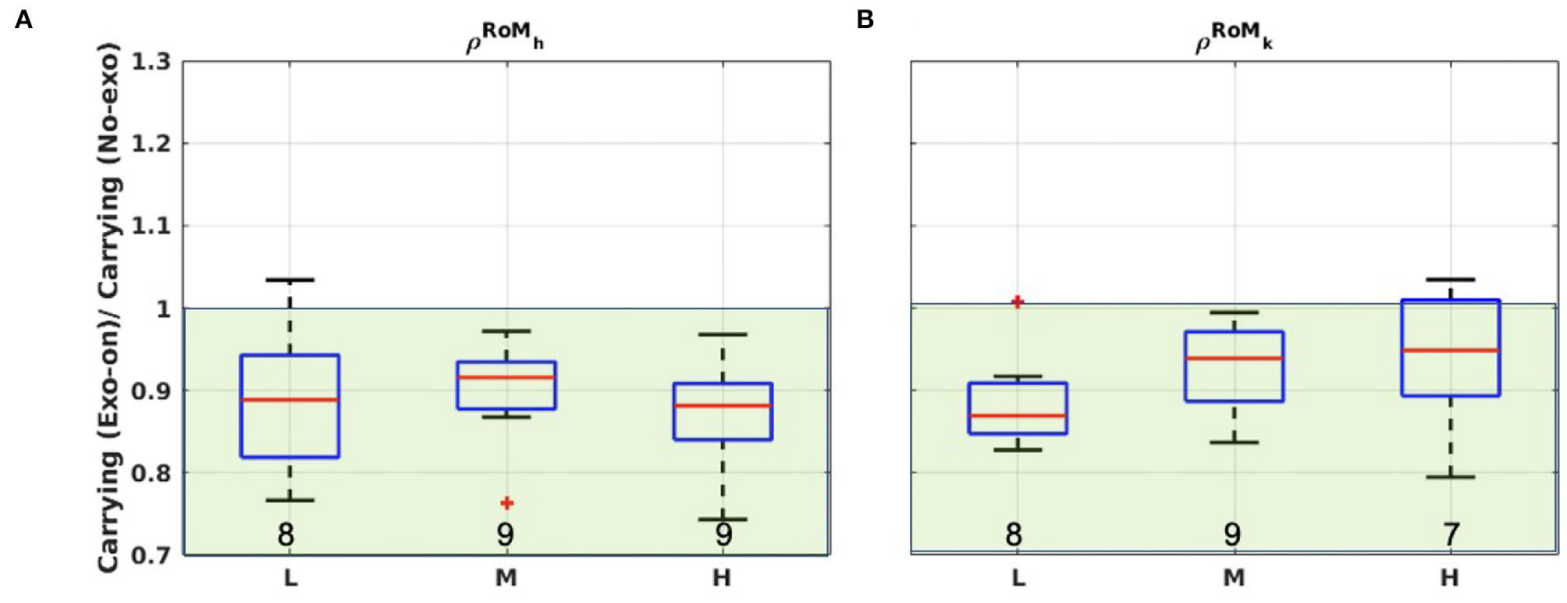
Boxplots representations for $\rho^{RoM_{h}}$ and $\rho^{RoM_{k}}$ considering carrying in *Exo-on* and *No-exo* condition. Two different joints are analyzed: **(A)** Hip and **(B)** knee. L, M, and H refer to the light, medium, or heavy loading condition. Green-shaded areas identify the regions where $\rho_{i}^{x}<1$ (i.e., the exoskeleton reduces the RoMs). Numeric values at the bottom of each box report the value of γ.

How the RoM changed between the two conditions is reported in Figure 6, revealing a clear trend for both the hip and the knee joints. Indeed, for both hip and knee RoM it almost always holds that $\rho^{RoM_{k}}<1$ and $\rho^{RoM_{h}}<1$ (see also γ values). On average, the median values (α) are around 0.90 indicating that there is a reduction in the RoMs of about 10% due to the exoskeleton action. The *iqr* values are much lower than in the muscle analysis (maximum *iqr* is 0.12 with respect to 0.71).

Significance levels obtained comparing the *Exo-on* and the *No-exo* condition are reported in Table 2. Bold values indicate where significance was reached (*p* < 0.05). In each condition, at least one joint had a significant RoM reduction between the test and control condition.

**Table 2:** Gait kinematics—Statistical significance for the considered loading conditions.

	***L***	***M***	***H***
Hip	0.0534	0.0970	**0.0396**
Knee	**0.0040**	**0.0441**	0.1582

*The table reports the p-values obtained from the one-way ANOVA test. Bold values indicate where statistical significance was met (p < 0.05)*.

**Figure 7:**
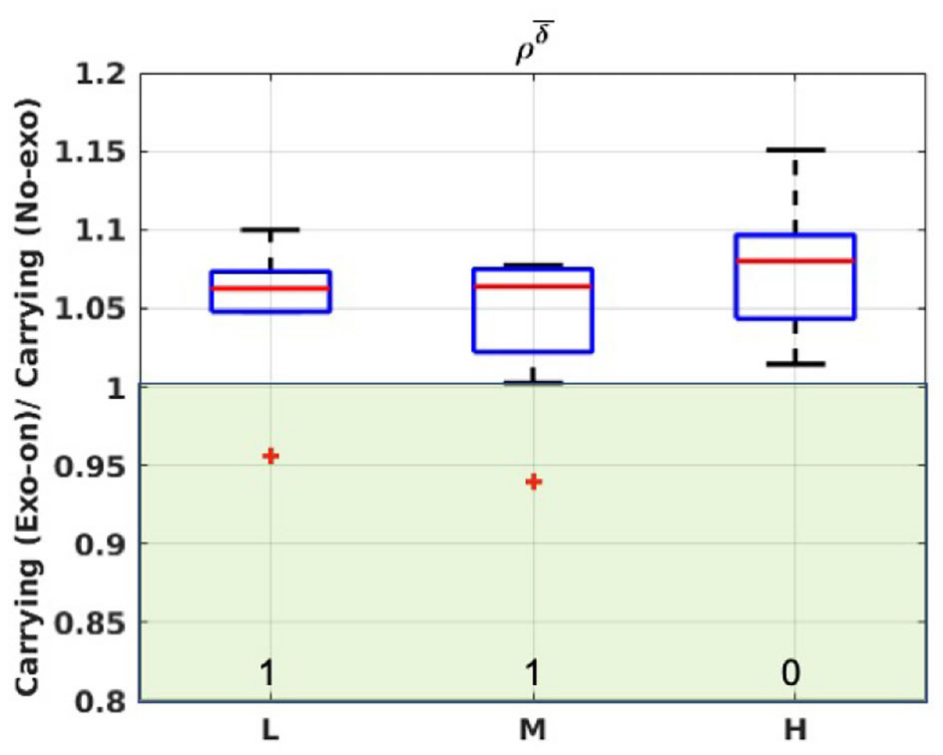
Boxplot representation for $\rho^{\bar{\delta }}$ considering carrying in *Exo-on* and *No-exo* condition. L, M, and H refer to the light, medium, or heavy loading condition. Green-shaded areas identify the regions where $\rho^{\bar{\delta }}<1$ (i.e., the exoskeleton reduces the stride duration). Numeric values at the bottom of each box report the value of γ.

Moreover, by inspection of Figure 7, it is possible to see how the *Exo-on* condition yielded an increase of stride duration ($\bar{\delta }$), as all the distributions lie in the $\rho^{\bar{\delta }}>1$ region. The trend indicates a median increase in cycle time duration of about 6%. Outliers can be identified in the light and medium load conditions. The values of γ indicate a clear effect for all the subjects.

#### 3.2.3. Subjective Perception

**Figure 8:**
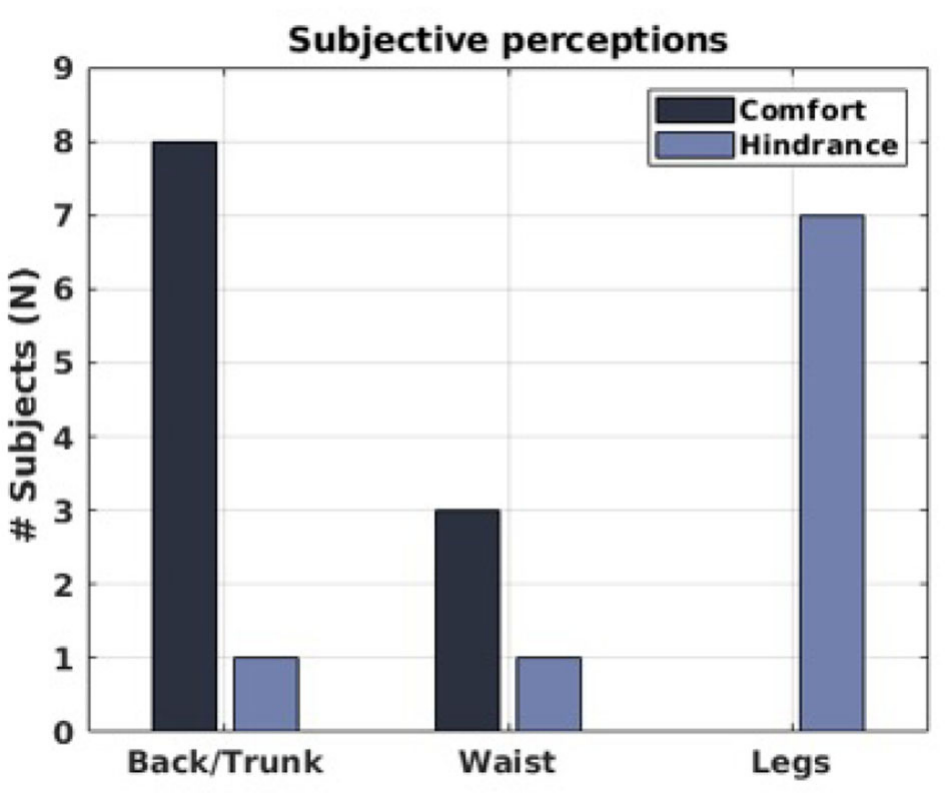
Subjective perceptions of the 9 users. For each of the considered body regions, it is reported how many users felt a benefit and how many experienced hindrance.

Finally, Figure 8 summarizes, for each body region under analysis, how many users reported a benefit or hindrance/discomfort when comparing the *Exo-on* and the *No-exo* conditions. The majority of the subjects (8 out of 9) experienced a positive exoskeleton effect on the back/trunk region, whereas 7 out of 9 subjects felt hindered in the lower limbs. Interestingly, 3 users reported benefit also on the waist, where the exoskeleton is anchored. As the users were instructed to report benefit or hindrance only if actually perceived, for a given body region, the sum of hindrance and benefit does not have to be *N* = 9.

## 4. Discussion

In the following, the discussion is presented starting from the analysis of the carrying impact on spinal loading compared to the lifting case. The authors' assumption was that such loading would be comparable between the two activities. This supports and validates the assertion that an occupational back-support exoskeleton is needed/valuable in providing assistance during carrying. Therefore, the first assessment is followed by an evaluation of the effects of an exoskeleton assisting with carrying while applying a constant extension torque provision. A control strategy of this type is a simplification of what happens if an exoskeleton, programmed to assist lifting, is also used during carrying. Here, the authors' hypothesis was that for carrying this strategy would turn out to be sub-optimal, namely being beneficial for the back but hindering the lower limbs. As a consequence, Section 4.3 focuses on the need to implement back-support exoskeleton versatility. Finally, the limitations of this study are discussed in Section 4.4.

### 4.1. The Impact on Spinal Loading

The results summarized in Figure 3 confirm that—from an ergonomic viewpoint—carrying activities can be associated with risk. Indeed, compared to lifting, muscle activity, although less during carrying, is in the same order of magnitude. In particular, as the handled payload increases, the differences between lifting and carrying are reduced and become less pronounced. This trend is particularly evident if the 50th percentile of the muscle activity distribution is considered. Generally, this value can be associated with repetitive/cumulative fatigue, whereas the 90th percentile is related to traumatic damages of the inter-vertebral discs. Despite traumatic damage is seen as more concerning, it is clear that damage can occur in both lifting and carrying and, thus, should be prevented/limited. The results found in section 3.1 are consistent with the ISO 11228-1 standard that establishes ergonomic guidelines for performing both lifting and carrying, identifying the latter activity as equally worthy of attention.

Therefore, it makes sense to try to assist also the carrying activities by means of an active occupational exoskeleton.

### 4.2. The Effects of Assistance During Carrying

The analyzed assistance principle implies that the delivered torques simultaneously support the extension of back and both hips. It was assumed that such assistance would be beneficial for the back, whereas it might hinder the natural movement of the hips, particularly in the swing phase.

The following discussion is separated according to the two body regions under analysis.

#### 4.2.1. The Lower Back

The experimental results do not indicate a clear polarity on the data and, thus, it is not possible to confidently conclude that, with respect to the conditions of this study, the exoskeleton is providing a reduction in the activation and work intensity of the lower-back muscles. Nevertheless, it is worth highlighting how the data variability shows a general trend to reduce as the payload increases and that the heavy load condition has a much clearer trend toward the ρ^*M*^, ρ^*P*^ < 1 region, i.e., where the exoskeleton has a benefit on the muscle activation. This suggests that conclusions drawn for this condition are more reliable than those drawn for lighter loads. In particular, for the heavier load condition, α values for ρ^*M*^ and ρ^*P*^ suggest that the exoskeleton effect is beneficial, reducing the overall lumbar activity by 12.08 and 7.99%, respectively. It is also important and encouraging that the exoskeleton is seen to have the greatest effect with the heaviest loading, as this is the circumstance that is most in need of assistance. Also, a comparison of objective and subjective data confirm the beneficial effect of the exoskeleton. Indeed, as outlined in Figure 8, 8 out of 9 users reported benefit on the back and only 1 out of 9 reported discomfort or hindrance on the same body segment. On the other hand, a part from the light load condition, lower-back muscle analysis showed that, out of 9, 5–6 subjects (according to the analyzed metric) had a reduction of muscle activation (see γ values in Figure 4). These values are not so far from those reported by the subjective evaluation forms. Therefore, the consistency between objective and subjective data suggest that, considering spinal loading, there is some evidence that the exoskeleton effect is somehow beneficial for most of the population.

Contrary to the authors' expectations, for the medium and high payload handling, the overall lumbar activity reduction is not in line with the potential of the device used in the assessment. In particular (Toxiri et al., 2018), the experiment showed significant back muscle activation reduction (around 30%), whereas a clear reduction was not obtained in this study, even though sound bio-mechanical models supported the authors' hypothesis. This, along with the negative exoskeleton effect for the light load condition, suggests that there is room for improving the constant torque strategy used in this study.

One upgrade is to modulate the delivered torque according both to the handled payload and to the user's body mass. In particular, the analysis of both α and γ supports the need to modulate the assistance according to the handled payload. Indeed, considering the lightest loading condition, the exoskeleton does not clearly reduce spinal loading, as highlighted by α. On the other hand, as the handled weight increases, the exoskeleton assistance results in reductions of α values both for ρ^*M*^ and ρ^*P*^ (α < 1). To this extent, it is interesting to note that, even if very slightly, the intermediate condition seems to be a minimum and might indicate that the amount of assistance provided is best around that payload range. Moreover, the number of subjects that show a benefit from the additional torque provided by the exoskeleton increased as the weight of the carried load increased (see Figure 4). For the light payload test, the muscle activity increase may be interpreted by subjects adopting abdominal and back-extensors co-contraction, stiffening the upper body to counteract the backwards push of the exoskeleton and to regain stability. Further experimentation could help clarifying this phenomenon and if modulating the torque according to the payload would, as expected, reduce it. Finally, the large variability in the results further suggests the possibility of modulating the delivered torque not only according to the payload, but also to the subjects' body mass. Indeed, despite body masses variability (76.55±8.22 kg), the delivered torque was kept constant, and so, it is possible that subjects with different body mass experience and react differently to the same amount of assistance.

#### 4.2.2. The Lower Limbs

The exoskeleton assistance on the lower limbs resulted in hindrance clearly affecting the gait kinematics of all the users. This hindrance is evident both as subjective perception of the users (7 out of 9 users felt hindered on the legs) and from the kinematic analysis. Indeed, hip and knee RoMs were reduced by up to 12%. Stride speed was also reduced due to a corresponding increase in stride duration (between 6 and 8%). In addition, a study conducted on the effects of load carriage on energy cost of walking (Abe et al., 2004) showed no significant differences in the energy cost associated with walking for values between the control condition (empty backpack) and the test one (backpack with a 6 kg load). This suggests that the differences noted in this study are related more to the exoskeleton torque provision, rather than the exoskeleton weight itself (6 kg). These elements suggest that simultaneously pushing both hips toward extension appears not to be the best assistive strategy.

Furthermore, although the kinematic analysis and the subjective perceptions are clearly polarized, this does not happen in the muscle analysis. There may be two main reasons that explain this lack of a clear trend.

The first reason being the non-ideal choice of the muscles. Indeed, partially due to the exoskeleton fitting and partially due to the difficulty in assessing via sEMG the hip flexor activity, in the proposed protocol it was not possible to measure the muscle activity of the Iliopsoas (hip flexor) and of the Gluteus Maximus (hip extensor) (Byrne et al., 2010). For these reasons, the activities of the RF and ST were chosen as representative of hip flexion/extension muscle activation. Problems in assessing the proper flexors and extensor are reported also in Baltrusch et al. (2019), where muscle activity did not show any significant differences between conditions.

The second reason why no trend emerges in the selected muscles might be related to the changes in the gait trajectory, as reported above. Analyzing both hip and knee joints, for each load condition, the exoskeleton assistance resulted in a reduced RoM. Indeed, almost all of the population lies in the $\rho^{RoM_{h}},\rho^{RoM_{k}}<1$ region. It is interesting to note the little data variability (*iqr*), suggesting its reliability.

Moreover, the one-way ANOVA test, significance level = 0.05, conducted to compare the *Exo-on* and the *No-exo* condition, found statistically significant differences at least for one joint in all of the conditions (see Table 2). In the case of the hip joint, the RoM reduction is due both to smaller flexion angles, hindered by the constant torque, and to smaller extension angles, possibly due to a compensation for the unwanted/unexpected backwards push of the exoskeleton. Differences in the knee trajectory can be explained as a consequence of the hip changes. Delving a bit more into the kinematic analysis, Figure 7 shows that the *Exo-on* condition caused a speed reduction in the users walking speed: all the population, apart from an outlier, lies within the $\rho^{\bar{\delta }}>1$ region. Therefore, reduced RoMs and slower stride durations show an evident hindrance confirming the authors' expectations.

### 4.3. On (the Need of) Back-Support Exoskeleton Versatility

To fully exploit back-support exoskeleton versatility, the standard control strategies can be expanded by including task awareness. This implies that, at first, the activity being performed by the user is recognized (high level), then, in accordance with the task the appropriate assistive strategy is selected (mid-level) and, finally, actuators are controlled to ensure that the provided torque is properly delivered (low-level). Such a distinction of control levels was presented in Tucker et al. (2015).

Now that data have been presented and discussed, there are more elements to debate on the need to recognize different tasks and the opportunity of selecting the controller according to the one being performed. Passive exoskeletons, generally lighter, simpler, and cheaper than active ones, can avoid the lower limb hindrance found in walking activities (Baltrusch et al., 2019). This is achieved by resorting to manual clutches, spring offsets, and automatic engage or dis-engage of passive elements, like in the commercial products by Laevo^2^ and Ottobock^3^, or in research prototypes (Jamšek et al., 2020). On the other hand, due to mechanical design limitations, passive devices cannot provide support in carrying activities. This means that there is no need to discriminate among *walking* or *carrying*.

Unlike passive devices, active exoskeletons are more versatile and, so, are able to exploit the functionality and flexibility of their actuators to create assistance profiles that can be tailored to the demands of the assistive task, like carrying in this case. Not all the active exoskeletons, however, have the same “*degree of versatility*.” As an example, the H-WEX exoskeleton presented in Ko et al. (2018) cannot provide support differently from the approach presented in this study. This is due to the choice of a single motor for the actuation, resulting in a more compact, efficient, and lightweight exoskeleton. However, the single motor can only modulate the delivered amount of torque and cannot assist the legs independently, according to gait phase. Instead, as an example, the APO exoskeleton (Chen et al., 2018) and the XoTrunk exoskeleton used in this study have two motors, one on each side. This design choice can be exploited to develop new assistance strategies, more appropriate for carrying. Indeed, in the previous sections, it has been discussed how a better strategy could improve the effectiveness of the exoskeleton for the back region and reduce the hindrance in the lower limbs (as seen in the data analysis). Hence, considering active exoskeletons, distinguishing among walking, carrying, and lifting is supported both by the relevance of carrying activities and by the need to switch between different controllers.

As a final comment, it is useful to note that in Poliero et al. (2019b) the distinction between lifting and walking only takes into account kinematic variables, whereas specific sources of information (like forearm muscle activity, sensorized gloves/insoles, or vision) are used to discriminate among walking and carrying. This final consideration highlights that not only mechanical choices but also control ones can affect the versatility of back-support exoskeletons.

### 4.4. Limitations

In the designed testing protocol, MVC calibration was performed adopting a single posture. However, this procedure is more prone to variability in the MVC normalization as subjects might exhibit differences in the posture to obtain maximum muscle activity (McGill, 1991). The large inter-subject variability did not allow us to always apply standard statistical analysis such as the analysis of variance. For this reason, the authors have decided to perform intra-subject normalization between the control and test conditions. As a consequence, the results are discussed taking into consideration trends. The proposed testing protocol was carried out in a lab setting. This might present substantial differences to the conditions found in a workplace where users may be required to walk on undulating or sloped surfaces in addition to level ground. Therefore, our findings cannot be directly generalized to out-of-the-lab scenarios. Additionally, the indication to perform the carrying task at a self-selected speed might be a further simplification of actual working conditions. Indeed, for given tasks, the workers could be required to walk as fast as possible so as not to limit productivity. Also, the relatively short duration of the activities performed during the testing protocol does not allow us to observe fatigue effects, or the effects of prolonged exoskeleton usage.

## 5. Conclusion

In the context of manual material handling and, more specifically regarding the ISO 11228-1 standard, carrying can have an impact on the spinal loading comparable to lifting. Back-support exoskeletons are generally used to assist lifting and, thus, mitigate the ergonomic risks associated with this activity. The applicability of these devices to other activities, such as carrying, is still an open issue.

This paper investigates first the effects of carrying on spinal loading and, then, the effects of assisting carrying with an exoskeleton designed for lifting tasks support. An experimental campaign involving 9 users and three different payloads (1.2, 8.2, and 16.2 kg) was designed to assess the relevance of carrying and the benefits arising from providing assistance for this task, in the same way it is done for symmetric lifting, i.e., synchronously supporting back and both hips extension.

The findings indicate that carrying, from an ergonomic viewpoint, is a relevant activity because the corresponding spinal loading is comparable to lifting.

Contrary to the expected outcome, the experimental results do not provide clear evidence on the effectiveness of the analyzed strategy in supporting the lower-back. However, the overall lumbar activity shows a promising trend when carrying heavy objects as for muscle activation is reduced by up to 12%. Large data variability invites caution when interpreting it. In agreement with the expectations, the strategy yielded hindrance for the lower limbs. This is supported by reduction in hip and knee RoMs (around 10%) and an increase of stride duration (between 6 and 8%). Due to changes in gait kinematics and difficulties in assessing the proper hip flexor and extensor, muscular analysis for the lower limbs did not provide significant findings.

Finally, there has been a discussion on how a better control strategy could improve the effectiveness of the exoskeleton. As control strategies for back-support exoskeletons start addressing tasks differing from lifting, the capability of recognizing which activity is being performed and, thus, triggering the appropriate controller, becomes a relevant feature, promoting active exoskeletons versatility.

## Data Availability Statement

The raw data supporting the conclusions of this article will be made available by the authors, without undue reservation.

## Ethics Statement

The studies involving human participants were reviewed and approved by Comitato Etico Regione Liguria. The patients/participants provided their written informed consent to participate in this study. Written informed consent was obtained from the individual(s) for the publication of any potentially identifiable images or data included in this article.

## Author Contributions

TP, ML, ST, CD, and JO devised the experiment. TP led the experiment and performed data analysis. ML and ST helped run the experiment. ST, ML, CD, and JO helped with data analysis. All authors contributed to the manuscript drafting. JO and DC obtained funding and resources.

## Conflict of Interest

The authors declare that the research was conducted in the absence of any commercial or financial relationships that could be construed as a potential conflict of interest. The reviewer MBN declared a past co-authorship with several of the authors (TP, MZ, ST, DC, JO) to the handling Editor.

## Footnotes

^1^protocol reference number: CER Liguria 001/2019.

^2^http://en.laevo.nl/

^3^https://paexo.com/paexo-back/?lang=en
